# Vinasse: from a residue to a high added value biopolymer

**DOI:** 10.1186/s40643-021-00476-1

**Published:** 2021-12-17

**Authors:** Daiana V. Trapé, Olivia V. López, Marcelo A. Villar

**Affiliations:** 1 Planta Piloto de Ingeniería Química, PLAPIQUI (UNS-CONICET), Camino La Carrindanga Km. 7, 8000 Bahía Blanca, Argentina; 2 Departamento de Ingeniería Química, Universidad Nacional del Sur, Av. Alem 1253, 8000 Bahía Blanca, Argentina; 3 Departamento de Química, Universidad Nacional del Sur, Av. Alem 1253, 8000 Bahía Blanca, Argentina

**Keywords:** Vinasse, Polyhydroxybutyrate (PHB), *Bacillus megaterium*, Microbial fermentation, Culture medium optimization

## Abstract

This work aimed to study the feasibility of using vinasse for polyhydroxybutyrate (PHB) production by *Bacillus megaterium*. To optimize the culture medium, a Box–Behnken design was employed considering carbon (C), nitrogen (N), and phosphorus (Ph) concentrations as independent variables and PHB productivity as the response variable. The productivity decreased when C or N were increased, probably due to the presence of phenolic compounds and the limitation of N for the production of PHB by *Bacillus* sp. bacteria. An additional experimental design to optimize the C/N ratio and growing conditions (fermentation time and temperature) was carried out. Fermentation time had a statistically significant effect on PHB productivity reaching 10.6 mg/L h. On the other hand, the variability in physicochemical properties of vinasse samples led to significant differences in PHB productivity. Lower productivity values were obtained when vinasse had higher values of DBO. Therefore, biopolymers production from vinasse is a feasible alternative to valorize this bioethanol by-product.

**Graphical Abstract:**

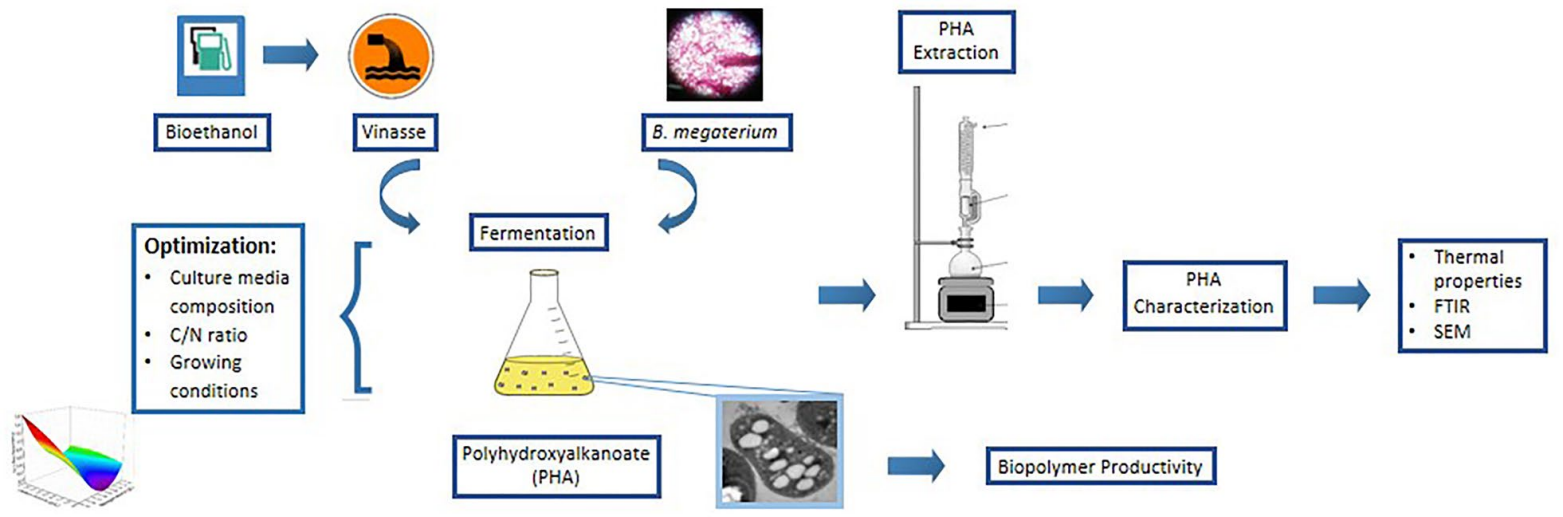

## Introduction

Plastics obtained from non-renewable sources are one of the most used materials in the world, they are broadly integrated into today’s lifestyle and contribute to almost all product areas. However, when plastics are discarded into the environment, they can persist for very long periods of time (Andrady 2015). For this reason, in the last years, alternatives have been explored to replace petroleum derived plastics by materials obtained from natural sources. Hence, several materials have been investigated such as biobased poly(ethylene terephthalate) (bio-PET), biobased poly(ethylene) (bio-PE), bio-poly(carbonate) (bio-PC), bio-poly(amide) (bio-PA), poly(hydroxyalcanoate)s (PHAs), and poly(lactic acid) (PLA), among others. PHAs are polyesters of hydroxyacids naturally synthesized by bacteria as carbon reserve. These biopolymers are accumulated as cytoplasmic inclusions in certain bacteria during unbalanced growth conditions, usually characterized by an excess of the carbon source and the lack of at least one of the essential nutrients (Kovalcik et al. 2019). Poly(3-hydroxybutyrate) (PHB) is the most common and the best known PHA and it is a great alternative to develop biomaterials since this biopolymer has similar properties to conventional polymers such as PE and poly(propylene) (PP) (Vandi et al. 2018). Also, PHB decomposes to water and carbon dioxide under aerobic conditions and to methane and carbon dioxide under anaerobic conditions by microorganisms in soil, sea, lake water, and sewage (Anjum et al. 2016). Its applications include packaging materials, bags, containers, sutures, cardiovascular stents, targeted tissue repair/regeneration devices, polymer-based depots for controlled drug release or implants, and disposable items like single-use cups and diapers (Koller 2018).

Despite these advantages, the high production cost of PHB is the main obstacle for its commercialization. PHB cost is at least three times higher than conventional plastics such as PP and PE, attributed mainly to the expensive substrates and processing (Kourmentza et al. 2017), and similar to biobased polymers such as PLA (Kaur et al. 2017). Thus, using cheaper feedstocks is one of the keys to reduce PHB production costs. Food wastes (Nielsen et al. 2017), residues from beer breweries (Amini et al. 2020), cheese whey (Pais et al. 2016), olive mill wastewater (Alsafadi and Al-Mashaqbeh 2017), and hydrolyzed corn starch (Fabra et al. 2016) are some resources that have been investigated for sustainable PHB production. On the other hand, vinasse, a residue of the sugar cane alcohol industry, could be used as an economic substrate to produce PHB at lower costs. In this sense, some authors have reported the use of vinasse as carbon source for PHB production. Bhattacharyya et al. (2012) used vinasse with *Haloferax mediterranei* to produce PHAs and they reported that concentrations higher than 10% of raw vinasse inhibited the microbial growth. On the other hand, Zanfonato et al. (2018) did fermentations with *Cupriavidus necator* using vinasse as carbon source and no inhibitory effect was observed. Pramanik et al. (2012) employed raw vinasse as carbon source to produce PHB with *Haloarcula marismortui* and achieved an accumulation of 23% PHA (of cell dry weight). These authors also stressed that after a pretreatment process through adsorption on activated carbon, vinasse could be used leading to 30% accumulation. In other cases, a combination of vinasse and sugarcane molasses was used by *Cupriavidus necator* as microorganism (Dalsasso et al. 2019; Acosta-Cárdenas et al. 2018). They showed that vinasse can be an appropriate diluent to molasses to use as non-conventional culture medium in the production of PHAs. Nowadays, vinasse disposal represents an environmental problem since it affects water and edaphic resources, as well as the life of animal and plant species. The high solids concentration and hard constituents, such as phenols and polyphenols, contaminate the surface and underground water. Also, vinasse presence increases eutrophication due to its high content of nitrogen and phosphorus (Parsaee et al. 2019). Colored compounds of vinasse reduce the permeability of sunlight in rivers and lakes, thereby reducing the photosynthetic activity and the concentration of dissolved oxygen in water generating disturbances of plants and aquatic animal life (Syaichurrozi 2016). Vinasse is also toxic because of its low pH, it has an unpleasant smell for humans and its influence on drinking water leads to an outbreak of malaria, amoebiasis, and schistosomiasis. It is a medium for worm eggs of *Meloidogyne javanica*, *M. incognita*, and *Drosophila melanogaster* to grow. The pollution of each liter of vinasse is equal to the amount of contamination produced by 1.43 humans (Parsaee et al. 2019).

Optimization of the fermentation process is another way to reduce the costs of PHB. The optimization by statistical methods, compared to the common “one factor at a time” method, proved to be a powerful and useful tool to predict the maximum yield for bioproducts synthesis. Statistical methods such as response surface methodology enable to design the experiments and to evaluate the interactions among factors and responses throughout the study (Nor et al. 2017). According to Nygaard et al. (2019), in the case of microbiological fermentation processes to produce PHAs, response surface methodology can be used to determine the composition of the culture medium that provides an optimal productivity of PHAs. Before using the response surface methodology approach, the correct experimental design must be selected to designate which treatments should be done in the experimental region being studied. For this purpose, experimental designs for quadratic response surfaces, such as three-level factorial, central composite, and Box–Behnken, should be applied (Yolmeh and Jafari 2017). A comparison between the Box–Behnken design and other response surface designs (central composite and three-level full factorial design) had demonstrate that Box–Behnken design is slightly more efficient than central composite design, but much more efficient than the three-level full factorial designs (Ferreira et al. 2007). For these reasons, Box–Behnken design was selected for this work.

In the present work, PHAs were obtained by *Bacillus megaterium* employing vinasse as carbon source. A statistical experimental design was applied to optimize the medium composition and fermentation conditions at laboratory scale. Synthesized biopolymers were extracted from the cells and characterized to evaluate their composition and thermal properties.

## Materials and methods

### Bacterial strain

Fermentations for PHAs production were carried out with *Bacillus megaterium* (GenBank Database Accession Number: HM119600.1), named as BBST4. This strain, isolated from sediments of the Bahía Blanca Estuary, was identified and characterized by López et al. (2012). *Bacillus megaterium* was adapted to metabolize vinasse by several fermentations increasing gradually the vinasse concentration. Adapted bacteria were conserved at − 70 °C in vials containing 1 mL growth medium and 20% glycerol. The growth medium was composed of 10 g/L vinasse, 10 g/L yeast extract, and 5 g/L peptone. Bacteria cells were reactivated at 30 °C for 24 h in the described growth medium. They were stored at 4 °C in slopes containing the growth medium and 20 g/L agar. Fermentation inocula were prepared by transferring bacteria cells using a wire loop into a shake flask containing 50 mL of the growth medium that were cultivated at 30 °C for 24 h.

### Carbon source

**Fig. 1:**
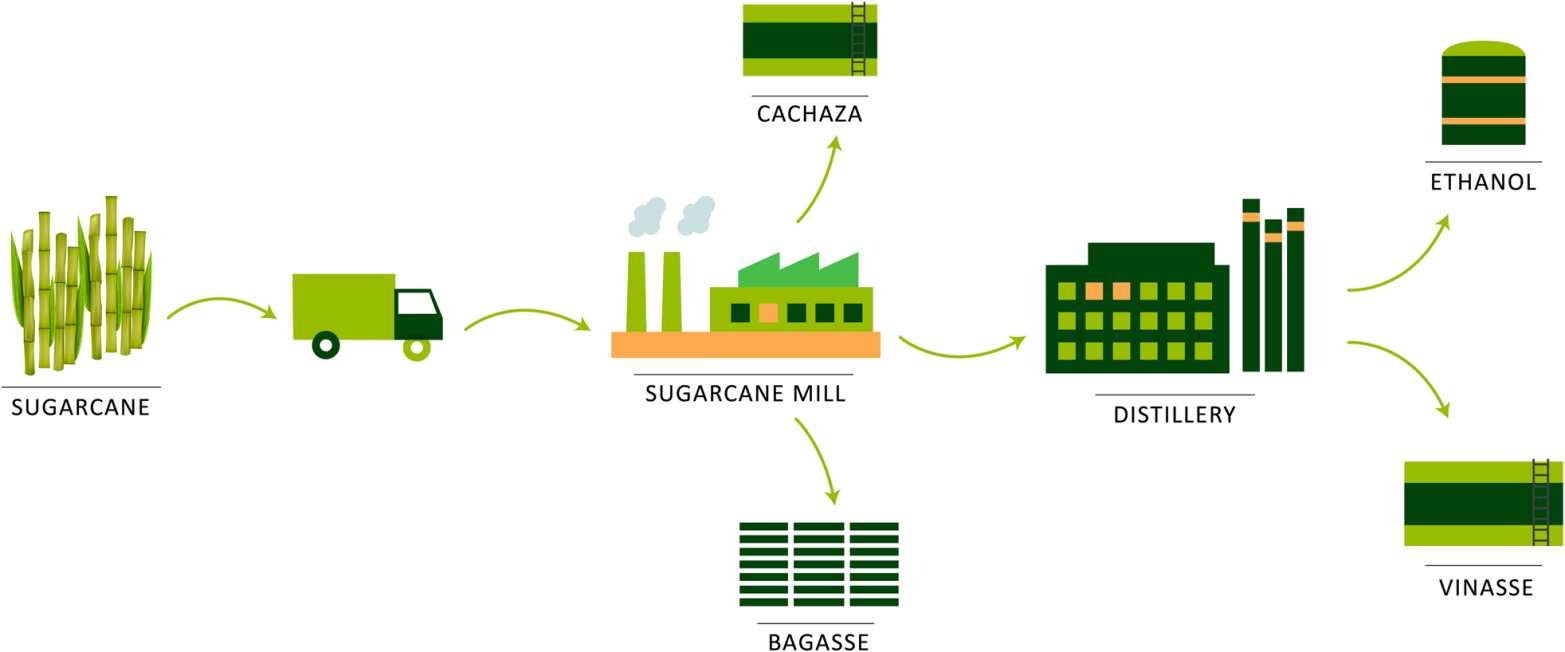
Flowchart of bioethanol production process

Vinasse, provided by a sugar-alcohol company located at Famaillá (Tucumán, Argentina), was used as carbon source. In Fig. 1 it is shown the flowchart of the bioethanol process from sugar cane. As it can be observed, vinasse is one of the byproducts resulting from this process. In this work, two vinasse samples called V_2017_ and V_2018_ were used.

Vinasse samples were physicochemically characterized by chemical oxygen demand (COD), biological oxygen demand (BOD), total organic carbon (TOC), total nitrogen (TN) and pH. Many of these studies were carried out at the Environmental Chemistry Laboratory, Universidad Nacional del Sur (Bahía Blanca, Argentina) by different standard methods: TOC was determined using a TOC analyzer (Exeter analytical CE440); TN by the Kjeldahl method (Bradstreet 1954); TSS by the Standard Method 2540 D (Total Suspended Solids Dried at 103–105 °C); TFS and TVS by the Standard Method 2540 E (fixed and volatile solids ignited at 550 °C), using the protocols described by Standard Methods for the examination of Water and Wastewater 20th Edition (Eaton et al. 1998); COD by the Standard Method Potassium Dichromate (Burke and Mavrodineanu 1977); and BOD using the Winkler method (Prambudy et al. 2019). The pH was measured using a Sensorex pH meter.

### Culture media

The composition of the culture media was: 2.5 g/L MgSO_4_·7H_2_O, 2.5 g/L NaCl, 0.5 g/L FeSO_4_·7H_2_O, 0.05 g/L MnSO_4_·4H_2_O, vinasse, NH_4_NO_3_, and K_2_HPO_4_. Concentration of carbon, nitrogen, and phosphorous was achieved varying the quantities of vinasse, NH_4_NO_3_, and K_2_HPO_4_ added to the culture medium. The pH of the media was adjusted to 7.0 by adding dilute sodium hydroxide solution.

### Optimization of culture media composition and fermentation conditions

For the optimization of culture media composition and fermentation conditions, a Box–Behnken experimental design and response surface methodology was employed.

To optimize the composition of the culture media, three independent variables were chosen: carbon (C), nitrogen (N), and phosphorus (Ph) concentrations, which were prescribed into 3 levels code: − 1, 0, 1 (Table 1). The ranges of the concentrations of C, N, and Ph were selected based on previous works (Acosta-Cárdenas et al. 2018; Wang et al. 2013; Berekaa and Al Thawadi 2012). The design matrix for 15 experiments is presented in Table 2. The response variable was PHB productivity (expressed in mg/L h). In the experimental assays, cultures were incubated in 250-mL Erlenmeyer flasks, containing 100 mL of each culture medium given by the experimental design, at 30 °C for 24 h and 150 rpm in an orbital shaker.

**Table 1:** Independent variables with their respective coded values and levels used in the Box–Behnken design

Culture media composition			
Level	C (g/L)	N (g/L)	Ph (g/L)
High (+ 1)	8	0.4	1.2
Central (0)	6	0.25	1.05
Low (− 1)	4	0.1	0.9

Fermentations conditions			
Level	C/N	*T* (°C)	*t* (h)
High (+ 1)	40	37	72
Central (0)	30	33.5	48
Low (− 1)	20	30	24

*C* carbon; *N* nitrogen; *Ph* phosphorous; *C/N* carbon/nitrogen ratio; *T* fermentation temperature; *t* fermentation time

**Table 2:** Factor values for independent variables and response variable (experimental and predicted) of screening experiments to optimize culture media composition

Run	C (g/L)	N (g/L)	Ph (g/L)	*P—*experimental (mg/L h)	*P—*predicted (mg/L h)
1	− 1	− 1	0	1.75	1.80
2	1	− 1	0	0.54	0.69
3	− 1	1	0	0.59	0.60
4	1	1	0	0.24	0.44
5	− 1	0	− 1	0.25	0.64
6	1	0	− 1	0.46	0.67
7	− 1	0	1	1.29	1.24
8	1	0	1	0.26	0.03
9	0	− 1	− 1	1.55	1.33
10	0	1	− 1	1.38	1.11
11	0	− 1	1	1.52	1.77
12	0	1	1	0.43	0.64
13	0	0	0	0.27	0.29
14	0	0	0	0.27	0.29
15	0	0	0	0.32	0.29

*C* carbon; *N* nitrogen; *Ph* phosphorous; *P* PHB productivity

To complement the optimization of PHA production by *B. megaterium* and vinasse as carbon source, a second experimental design was carried out. As independent variables were chosen carbon/nitrogen ratio (C/N), temperature (*T*), and time (*t*) and they were prescribed into 3 levels code: − 1, 0, 1 (Table 1). The ranges of C/N ratios, *T*, and *t* were selected based on previous works (Pal et al. 2009; Grothe et al. 1999). The design matrix for 15 experiments is presented in Table 3. As in the case of optimization of culture media, the response variable was PHB productivity. Cultures were incubated in 250-mL Erlenmeyers, containing 100 mL of culture medium with different C/N ratios at different temperatures and times, following the experimental design.

**Table 3:** Factor values for independent variables and response variable (experimental and predicted) of screening experiments to optimize fermentation conditions

Run	C/N	*T* (°C)	*t* (h)	*P—*experimental (mg/L h)	*P—*predicted (mg/L h)
1	− 1	− 1	0	5.1	5.3
2	1	− 1	0	5.5	4.6
3	− 1	1	0	4.4	5.3
4	1	1	0	4.0	3.9
5	− 1	0	− 1	9.7	9.9
6	1	0	− 1	4.0	5.3
7	− 1	0	1	3.0	1.8
8	1	0	1	4.5	4.4
9	0	− 1	− 1	10.3	10.0
10	0	1	− 1	9.5	8.5
11	0	− 1	1	3.2	4.3
12	0	1	1	4.8	5.2
13	0	0	0	6.9	6.6
14	0	0	0	6.3	6.6
15	0	0	0	6.5	6.6

*C/N* carbon/nitrogen ratio; *T* fermentation temperature; *t* fermentation time; *P* PHB productivity

After all fermentation assays, culture medium was centrifuged for 10 min at 4000 rpm. Cell pellets were washed with distilled water 3 times and dried to constant weight at 60 °C in an oven with forced air circulation.

### Quantification of accumulated PHA

Gas chromatography (GC) was used to quantify accumulated PHA. This technique specifically determines PHB. Samples for GC analysis were prepared as described by Riis and Mai (1988). Two mL of 1,2 dichloroethane, 2 mL propanol containing hydrochloric acid (1 volume concentrated hydrochloric acid  + 4 volume propanol) and 200 μL of internal standard solution (2 g benzoic acid in 50 mL propanol) were added to 40 mg of dry bacterial mass. Then, the sample was kept for 2 h in an incubator at 100 °C. After cooling to room temperature, 4 mL of water were added, and the mixture was shaken for 20–30 s. The heavier phase was injected into a gas chromatograph Hewlett Packard 5890 (Series II).

To quantify PHB, a calibration curve was previously performed by using a commercial PHB (Biomer, Germany). Productivity (*P*) of PHB was calculated using Eq. 1:

1 $$P = \frac{{{\text{PHB}}}}{t V},$$

where *PHB* is the quantity of the biopolymer in milligrams*, t* is the fermentation time in hours, and* V* is the culture media volume in liters*.*

### PHA extraction

Centrifuged cells were lyophilized in a Rificor, L-A-B3-C lyophilizer and PHA was extracted with chloroform in a Soxhlet apparatus during 48 h. After extraction, solvent was removed by evaporation and PHA yield was calculated by Eq. 2:

2 $${\text{PHA}}_{{{\text{yield}}}} = \frac{{{\text{PHA}}_{{{\text{extracted}}}} }}{{{\text{Dry}}\, {\text{cell}} \,{\text{weight}}}} \times 100,$$

where PHA_extracted_ corresponds to the PHA obtained after solvent extraction and dry cell weight is the amount of lyophilized cell used, both expressed in g/L.

### Characterization of extracted PHA

#### Fourier-transform infrared (FTIR)

FTIR analysis was performed using a spectrophotometer Nicolet Nexus. Samples were well-mixed with KBr (Sigma-Aldrich, 99% purity) at 1% w/w and press in order to obtain transparent discs. Spectra were obtained from 100 accumulated scans at 4 cm^−1^ resolution in the range 4000–400 cm^−1^.

#### Scanning electron microscopy (SEM)

Scanning electron microscopy (SEM) was conducted on a JEOL JSM-35CF electron microscope at an accelerating voltage of 10 kV. Samples were dispersed over 3M aluminum conductive tape stuck onto stubs by using an air flow and coated with gold in a sputter coater SPI.

#### Differential scanning calorimetry (DSC)

Thermal properties were evaluated by differential scanning calorimetry (DSC) using a Perkin-Elmer DSC calorimeter under nitrogen atmosphere. Analysis was carried out on  ~ 8 mg of sample heating from 25 to 190 °C, followed by a subsequent cooling down to 25 °C, and finally a second heating to 190 °C. All thermal ramps were performed at a rate of 10 °C/min. Melting point (*T*_m_) and melting enthalpy (*∆H*_m_) were obtained from thermograms at the second heating stage.

#### Thermogravimetric analysis (TGA)

Thermal degradation was performed using a thermogravimetric balance Discovery TA Instruments. Samples (~ 8 mg) were heated from 25 to 700 °C, under nitrogen atmosphere, at 10 °C/min. Mass–temperature curves were obtained and the first derivative was calculated. The peak of the first derivative indicates the maximum thermal degradation temperature (*T*_d_), point of maximum rate of change on the mass curve.

## Results and discussion

### Carbon source

Vinasse samples were physicochemically characterized and results are shown in Table 4. As it can be observed, physicochemical parameters of both samples (V_2017_ and V_2018_) were significantly different between them. This variability depends on the sugar cane variety and maturation, the substrate used in fermentation, distillation, and sulfitation process that enriches the downstream products with sulfur compounds especially sulfate species (Godoi et al. 2019). This wide range of physicochemical properties represents a challenge to use vinasse as carbon source on PHA production. Even though both studied vinasse samples contained raised BOD and COD levels and high suspended solids, V_2017_ presented significantly higher values than V_2018_. These parameters are responsible for the difficult disposal and are according to the average values found in the literature (Acosta-Cárdenas et al. 2018; Carrilho et al. 2016; Nakashima and Oliveira 2020). TN content of V_2017_ was 4.3 times higher than the corresponding to V_2018_. In the bibliography it was found TN values goes from 60 mg/L (Zanfonato et al. 2018) to 587 mg/L (Popolizio 2017), demonstrating once again the variability of this by-product and the challenge when using it as carbon source to produce biopolymers via microbial fermentation. Regarding TOC content, the value corresponding to V_2018_ was about half of the content obtained for the V_2017_ sample. Parsaee et al. (2019) reported a TOC value of 20.16 g/L, similar to the value obtained for V_2018_. On the other hand, Fagier et al. (2018) obtained a TOC value of 48 g/L, similar to the value obtained for V_2017_. pH values of vinasse samples are in the range reported on the bibliography (Fukushima et al. 2019). These low values hinder the disposal of this by-product.

**Table 4:** Physicochemical properties of vinasse samples

Physicochemical property	Vinasse sample	
	V_2017_	V_2018_
BOD (mg/L)	96,500 ± 500	36,170 ± 1808
COD (mg/L)	1,01,600 ± 500	40,880 ± 822
TN (mg/L)	1100 ± 20	255 ± 10
TOC (g/L)	40.5 ± 0.5	16.3 ± 0.5
pH	4.8	4.6

*BOD* biological oxygen demand; *COD* chemical oxygen demand; *TN* total nitrogen; *TOC* total organic carbon

### Optimization of culture media composition and fermentation conditions

In this study, *Bacillus megaterium* was employed to synthesize biopolymers via microbial fermentation. According to López et al. (2012) and Porras et al. (2017), this strain is capable of producing PHB and PHA copolymers, respectively. To optimize culture media composition 15 experiments, given by the Box–Behnken experimental design, were carried out and obtained PHB productivity (*P*) values and predicted responses are included in Table 2. The vinasse sample employed in these assays as carbon source was V_2017_. The influence of the different tested independent variables (carbon, nitrogen, and phosphorus concentration) on the biopolymer productivity are given by the *p *value: those that presented *p*  < 0.05 significantly affected the response parameter, meanwhile variables that showed *p*  > 0.05 were considered not statistically significant.

*p* values for the three independent variables and their interactions are given in Table 5. It is important to note that not all variables affected the productivity in the same way. It can be seen that C and N statistically influenced PHB productivity, considering a significance level of 0.05 (*p*  = 0.0441 and 0.0280, respectively). On the other hand, phosphorus concentration was not statistically significant (*p * = 0.8941). Interactions between studied variables were not remarkable on PHB productivity, except the quadratic term *N*^2^ (*p*  = 0.0167).

**Table 5:** Coefficient and *p* values for independent variables and their interactions, obtained from optimization of culture media composition

Variable	Coefficient	*p* value
C	− 0.2956	0.044
N	− 0.3384	0.028
Ph	− 0.0155	0.894
C^2^	− 0.0783	0.651
C–N	0.2130	0.231
C–Ph	− 0.3106	0.104
N^2^	0.5745	0.017
N–Ph	− 0.2287	0.203
Ph^2^	0.3570	0.080

*C* carbon; *N* nitrogen; *Ph* phosphorous

For predicting the optimal culture media composition, a polynomial function was fitted to the experimental data (Eq. 3):

3 $$P = 0.286 - 0.296{\text{C}} - 0.338{\text{N}} - 0.010{\text{Ph}} - 0.080{\text{C}}^{2} + 0.213{\text{CN}} - 0.310{\text{CPh}} + 0.570{\text{N}}^{2} - 0.228{\text{NPh}} + 0.357{\text{Ph}}^{2} ,$$

where *P* is PHB productivity (mg/L h), meanwhile C, N, Ph represents the carbon, nitrogen, and phosphorus concentration (mg/L) in the culture media.

This polynomial function represents a good fit of experimental data since the determination coefficient (*R*^2^) was 0.8924. According to Nygaard et al. (2019), the model has a high correlation because *R*^2^ is in the range of 0.7–0.9. C and N variables have similar negative standardized coefficients (− 0.296 and − 0.338, respectively). The fact that these coefficients were negative means that PHB productivity decreased when C or N concentration were increased. Even though the coefficient of Ph was also negative, it was very low (− 0.010), indicating that the effect of this variable on PHB productivity was not significant. Quadratic terms are model fit coefficients which demonstrate that there is a curvature and a local optimum point could be found. Thus, the optimum level for each studied variable, estimated from the maximum point of the polynomial PHB model, was estimated using the solver function of Statgraphics Centurion XV.II X64 tools. Optimum level of C, N, and Ph were − 1, − 1 and 0.935, respectively. Comparison with data found in the bibliography is difficult because several factors should be taken into account to analyze the effect of these variables on PHB productivity. For example, Bora (2013) studied the PHA synthesis by *Bacillus megaterium*, using fructose as carbon source, and K_2_HPO_4_ and Na_2_HPO_4_ as phosphorus sources. This author reported that fructose and its interaction with Na_2_HPO_4_ significantly affected PHB productivity. On the other hand, Nygaard et al. (2019) carried out fermentations for PHA production using *Cupriavidus necator*, and evaluated the effect of carbon, nitrogen and phosphorus concentration, as well as medium pH on the biopolymer productivity. Obtained results showed that productivity was statistically affected by N, pH, and C^2^. These examples from the literature demonstrate that the effect of fermentation variables on PHA productivity depends on many factors such as strain, nutrient sources, and fermentation conditions, among others. Therefore, the comparison with results obtained in this work is not suitable.

**Fig. 2:**
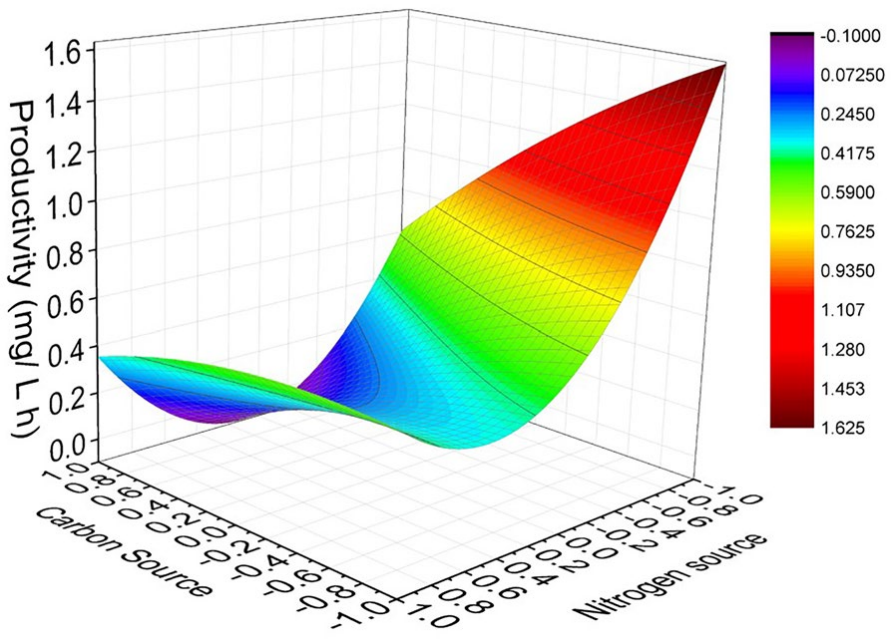
Response surface representing the effect of carbon and nitrogen concentration in culture medium on PHB productivity

As it was aforementioned, phosphorus concentration did not have a significant effect on PHB productivity, so the response surface was built taking into account only carbon and nitrogen concentrations as independent parameters and the productivity as response variable (Fig. 2). These response surface plots reinforced the discussed results about the effect of C and N concentrations on PHB productivity. As can be seen, productivity increased when the carbon and nitrogen concentration decreased. This could be attributed to the fact that vinasse contains phenolic compounds, difficult to be biologically degraded by bacteria, that have antimicrobial and phytotoxic properties (Parsaee et al. 2019). Besides, the decrease in PHB productivity by increasing nitrogen concentration may be due to that *Bacillus* sp. bacteria require the limitation of this nutrient for the production of PHB as metabolite (Kanekar et al. 2015).

From the optimum levels, PHB maximum productivity was calculated obtaining a value of 2.43 mg/L h. This is a low productivity compared to values found in the literature. For example, Bhattacharyya et al.(2012) reported a PHB productivity of 0.21 g/L h employing vinasse as carbon source and *Haloferax mediterranei* as strain. Therefore, to improve PHB productivity by *B. megaterium* employing vinasse as carbon source, an additional experimental design was carried out to optimize the C/N ratio and growing conditions (fermentation time and temperature). Therefore, 15 experiments given by the Box–Behnken experimental design were done and PHB productivity (*P*) values and predicted responses obtained are included in Table 3. The vinasse sample employed in these assays as carbon source was V_2018_. The *p *value for the three variables and their interactions are given in Table 6. Taking into account the different independent variables, fermentation time had a statistically significant effect on PHB productivity (*p*  = 0.0034). Meanwhile, fermentation temperature and C/N ratio did not significantly affect PHB productivity by *B. megaterium* (*p*  = 0.7016 and 0.2777, respectively). Regarding the interaction between the variables, the only one that had a notable influence on PHB productivity was the one between C/N and time (0.0318), probably due to the significant effect of fermentation time. None of the quadratic terms were statistically significant. Equation 4 described the polynomial model which was a good fit to the experimental data with a determination coefficient (R^2^) of 0.9093:

4 $$P = 6.57 - 0.524C/N - 0.175T - 2.25t - 1.7C/N^{2} - 0.2C/N.T + 1.8C/N.t - 0.08T^{2} + 0.6T.t + 0.5t^{2} ,$$

where *P* is the PHB productivity (mg/L h), *t* is the fermentation time (h), and *T* is the fermentation temperature (°C).

**Table 6:** Coefficient and *p* values for independent variables and their interactions, obtained from optimization of fermentation conditions

Variable	Coefficient	*p* value
C/N	− 0.5340	0.278
*T*	− 0.1878	0.702
*t*	− 2.2338	0.003
C/N-*T*	− 0.1580	0.756
C/N-*t*	1.8160	0.032
*T*–*t*	0.6016	0.370
(C/N)^2^	− 1.7675	0.495
*T*^2^	− 0.0898	0.901
*t*^2^	0.4981	0.495

*C/N* carbon/nitrogen ratio; *T* fermentation temperature; *t* fermentation time

**Fig. 3:**
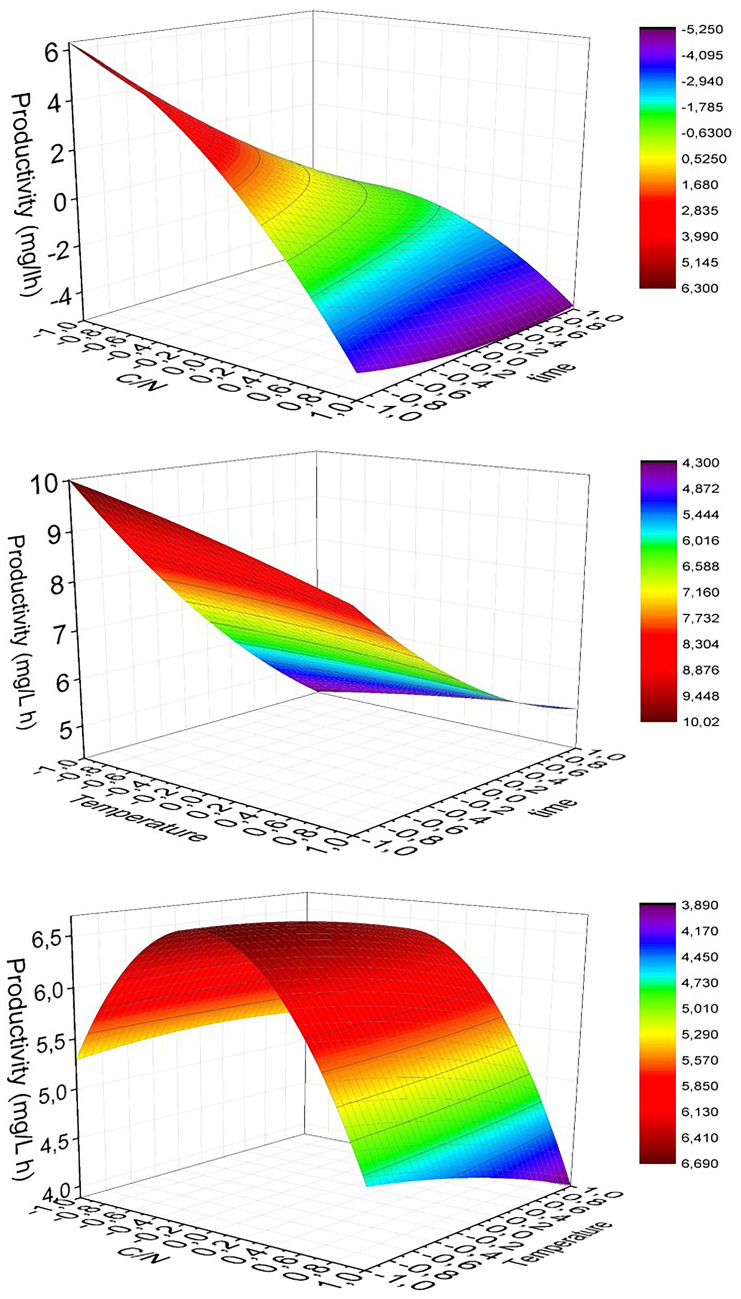
Response surface representing the effect of **a** C/N ratio and time, **b** temperature and time, and **c** C/N ratio and temperature on PHB productivity

The time variable had the largest negative standardized coefficient (− 2.25), indicating that an increase in the fermentation time led to a decrease in PHB productivity. The longer the fermentation time the lower the productivity. This tendency is in good agreement with the low PHB production rate, not accumulating more PHB until the end of the cultivation. Similar behavior was reported by Dalsasso et al. (2019) studying PHB production by *Cupriavidus necator* using a blend of vinasse and sugarcane molasses as substrate. On the other hand, coefficients of C/N and *T* variables presented very low values, demonstrating that they had no significant effect on the response variable. The quadratic coefficients and those of the interactions between variables resulted negligible, except the one corresponding to C/N and *t* interaction, mainly attributed to the effect of the fermentation time. Figure 3 shows the three response surface plots representing the productivity as response variable and C/N ratio and fermentation time (Fig. 3a), temperature and fermentation time (Fig. 3b), and C/N and temperature (Fig. 3c) as independent variables. The only significant interaction was between C/N and *t*; when *t* value was minimal and the ratio C/N increased, the production of PHB increased. Besides, when *t* reached its highest value, the slope of PHB production as a function of C/N became negative (Fig. 3a). The factor associated with the quadratic contribution of C/N presented its maximum value in the vicinity of the central point. Thus, the maximum PHB production occurred when C/N had a value of 23.95 (Fig. 3a, c). As it can be seen, the temperature range assayed in these experiences did not significantly affect the PHB productivity (Fig. 3b, c), despite these values being within the optimal growing temperature range for this strain (Porras et al. 2017).

The optimal value of C/N ratio, fermentation time and temperature to produce PHB by *B. megaterium* and vinasse as carbon source were 24.01, 30.25 °C and 24 h. The optimal productivity value in this case, predicted by the model, was 10.6 mg/L h. In order to verify the good correlation of the model, fermentations was carried out under these optimal conditions that theoretically maximize the PHB productivity and the experimental value resulted being 9.7 mg/L h. Thus, the effectiveness of the model was demonstrated since both values are similar with the same magnitude order. Pramanik et al. (2012) reported a similar PHB productivity (0.015 g/L h) using a culture medium with 10% raw vinasse as carbon source and *Haloarcula marismortui* as strain. Otherwise, reported values of PHB production by *Bacillus megaterium* by other authors are very variable according to the carbon source used. Jimenez (2011) informed a PHB productivity of 0.082 g/L h from glucose as carbon source, Obruca et al. (2011) reported a value of 0.056 g/L h employing cheese whey, and Porras (2012) obtained a PHB productivity of 0.0125 g/L h using starch.

As it was aforementioned, vinasse sample employed in the optimization of the media composition was V_2017_; meanwhile for the optimization of growing conditions, V_2018_ was employed. The variability in physicochemical properties of both vinasse samples led to significant differences in PHB productivity. As it can be seen, the best values were obtained using V_2018_ in the second experimental design. When V_2018_ was employed, it reached a productivity 4.4 times higher than value obtained with V_2017_, estimated for the optimal conditions. This difference could be associated with the higher BOD and COD values of V_2017_ than those of V_2018_. Particularly, BOD high values indicate that microorganisms need more oxygen to degrade it (Porras 2012) and this issue could affect the PHB production by *B. megaterium* from V_2017_.

### PHA extraction and characterization

Fermentations were carried out using the optimal composition. PHB was extracted and a characterization was done. Considering the mass of dry cells and extracted PHA, a polymer yield of 37% was obtaining. Valappil et al. (2007) reported a similar value for PHA yield (31%), using glucose as substrate and *Bacillus cereus* as microorganism. On the other hand, using vinasse as carbon source, Pramanik et al.(2012) obtained a PHA yield of 23% employing *Haloarcula marismortui* as strain and Zanfonato et al.(2018) reported a maximum PHA yield of 26% with *Cupriavidus necator*. In addition, *Bacillus *sp. accumulated 75.5% (Das et al. 2018), 54.6% (Mohanrasu et al. 2020) and 59% (Jimenez 2011) PHA using cheese whey in the first case and glucose in the others, respectively.

**Fig. 4:**
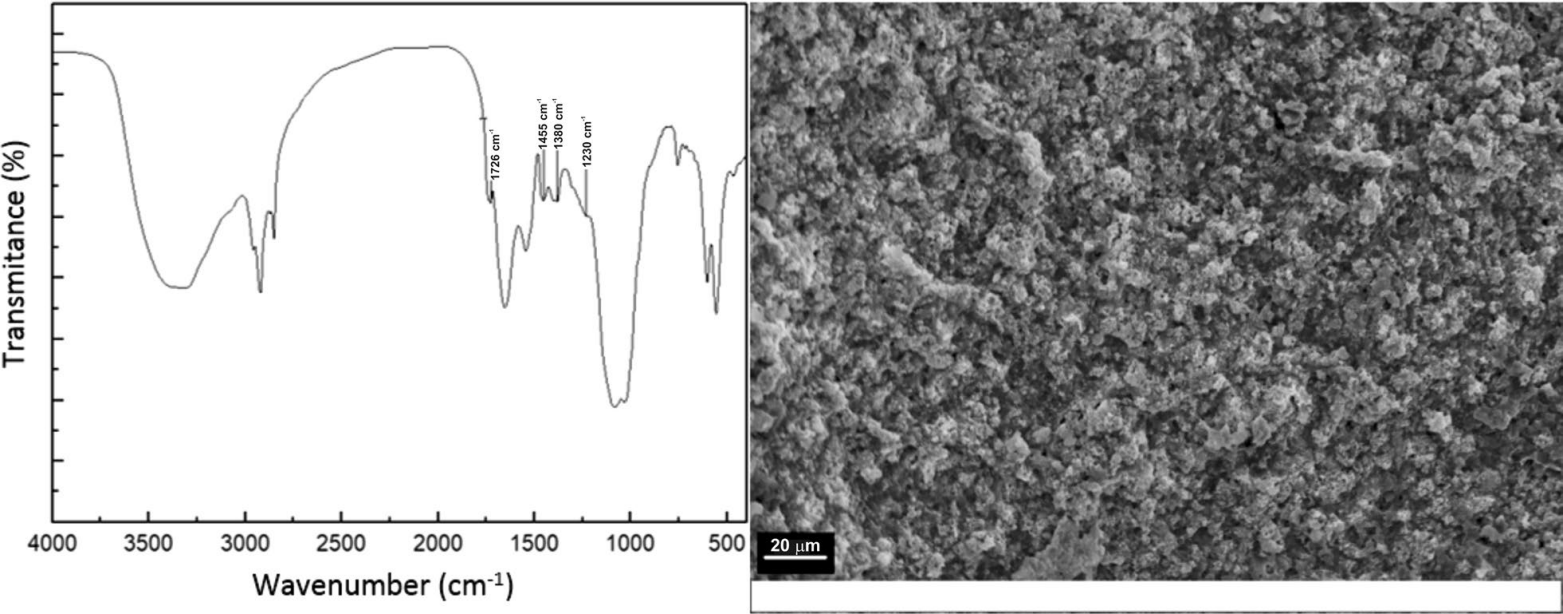
SEM micrograph and FTIR spectrum of PHA synthesized by *B. megaterium* using vinasse as carbon source

FTIR spectra of synthesized PHA are shown in Fig. 4 and it is in consonance with the PHB structure reported in the bibliography. A high intense band at 1726 cm^−1^ was observed which corresponds to the C=O stretch of the ester group (Lathwal et al. 2018). Absorption band at 1455 cm^−1^ is attributed to the asymmetric deformation of C–H bonds in –CH_2_ groups, while the band at 1380 cm^−1^ is assigned to the symmetric vibration of –CH_3_ groups. The band at 1230 cm^−1^ is assigned to CH_2_ vibrations (Jimenez 2011).

Figure 4 also includes a SEM micrograph of the extracted biopolymer, showing the morphology of PHA granules. The microstructure gives a fairly porous material with fine grains interconnected and a strong tendency to form multigrain agglomerates. The morphology shows grains that are pseudo-spherical in shape with fairly uniform distribution. Similar observations were reported by Nwinyi and Owolabi (2019). They used microbial species obtained from an abattoir employing different carbon sources (acetate and molasses) in the mineral medium.

The melting temperature of obtained PHB was taken at the maximum of the endothermic peak in the DSC second heating thermogram. A value of 177.7 °C was obtained which is in good agreement with values reported by Pradhan et al. (2018). These authors reported values of 175 °C and 176 °C for PHA obtained by *Bacillus megaterium* and *Cupriavidus necator*, respectively. The melting enthalpy determined of the PHA obtained in this study was 79.6 J/g. Pradhan et al.(2018) using *B. megaterium* and fructose as carbon source reported a melting enthalpy of 33 J/g and Ansari and Fatma (2016) obtained 83.2 J/g by *Nostoc muscorum* NCCU-442 and glucose as carbon source.

TGA was performed to detect the thermal stability of PHA. The maximum degradation temperature for the PHA synthesized was determined using the first derivative of thermogravimetric curve. PHA degradation occurred in two stages: in the first step, the degradation started at 225 °C and extended until 300 °C with a maximum degradation occurring at 255 °C and the second step began after 310 °C and the maximum degradation took place at 325 °C. The degradation in more than one stage was reported by various authors (Pradhan et al. 2018; Hassan et al. 2016; Liu et al. 2014). Particularly, Hassan et al. (2016) showed that PHB from *Bacillus* sp. was decomposed in 3 stages and resisted until 320 °C.

## Conclusions

A medium was optimized to maximize PHA production with vinasse as carbon source. It was possible to determine the optimal culture medium composition and operating conditions. Vinasse composition had an important effect on productivity. FTIR spectrum, SEM micrography and melting temperature of extracted biopolymer are in good agreement with values reported in the literature.

This study demonstrated the successful utilization of vinasse for PHB production by *Bacillus megaterium* at the shake-flask level. Although promising at the laboratory level, scaled-up fermentation studies with better controlled conditions (mainly pH and dissolved oxygen) can provide further insight into the functional feasibility of PHA production from vinasse.

## Data Availability

All data generated or analyzed during this study are included in the manuscript.

## Abbreviations

PHA: Poly(hydroxyalcanoate)s
COD: Chemical oxygen demand
BOD: Biological oxygen demand
TOC: Total organic carbon
TN: Total nitrogen
C: Carbon concentration
N: Nitrogen concentration
Ph: Phosphorus concentration
GC: Gas chromatography
PHB: Poly(3-hydroxybutyrate)
*P*: Productivity
C/N: Carbon/nitrogen ratio
*T*: Temperature
*t*: Fermentation time
FTIR: Fourier-transform infrared spectroscopy
SEM: Scanning electron microscopy
DSC: Differential scanning calorimetry
TGA: Thermogravimetric analysis
*T*_d_: Thermal degradation temperature
